# Broadening the spectrum of conflict and coexistence: A case study example of human-wolf interactions in British Columbia, Canada

**DOI:** 10.1371/journal.pone.0318566

**Published:** 2025-02-07

**Authors:** Ethan D. Doney, Beatrice Frank, Douglas A. Clark

**Affiliations:** 1 Department of Fisheries, Wildlife, and Conservation Sciences, Oregon State University, Corvallis, Oregon, United States of America; 2 Georgia Straight Alliance, Vancouver, British Columbia, Canada; 3 School of Environment and Sustainability, University of Saskatchewan, Saskatoon, Saskatchewan, Canada; University of Bucharest, ROMANIA

## Abstract

Coexistence has seen an explosive rise within conservation social science scholarship. While this represents an exciting shift in the field, many academics are still skeptical. Some scholars have expressed concerns around the omission of “conflict”, naïveté, and impracticality associated with coexistence literature. In this paper, we aim to demonstrate that critiques of coexistence often stem from reductionism and decontextualization, process inefficiencies and/or inequities, failure to address and prioritize human well-being as a goal, and a lack of tools to foster open, collaborative dialogue. We draw on a case study of human-wolf interactions in the Pacific Rim National Park Reserve Region, British Columbia, Canada, to illustrate how coexistence efforts can, and should, prioritize “conflict”, be attentive to the real challenges of sharing spaces with wildlife, and encourage collaborative, inclusive processes that work toward tangible, actionable outcomes. We conducted 32 semi-structured interviews with residents from diverse backgrounds and levels of experience with wolves in the region. From these interviews, we articulated novel, co-developed, contextual definitions of human-wolf conflict and coexistence in the region. We then developed a collaborative tool for visualizing behavioral and cognitive elements of human-wildlife interactions through open and inclusive dialogue, using real examples from these research interviews. The research findings highlight three main principles: (1) that conflict and coexistence are contextual and should be understood as such, (2) that coexistence requires collaborative processes that pay attention to equity and inclusivity, and (3) that there are frameworks or tools that can help facilitate discussions toward practical outcomes of coexistence projects. We believe that this paper helps to disambiguate coexistence and reinforce that coexistence requires focused attention to the well-being of people as much as wildlife.

## Introduction

The COP15 Global Biodiversity Framework includes formal wording and policy targets on human-wildlife conflict and coexistence [1]. This emphasis is likely tied to the rise in conservation social science research related to conflict, with a more recent focus on coexistence [2,3]. While the growing focus on coexistence is positive in theory, there have been expressed concerns related to the practicality of the term, the perceived naïveté of coexistence literature, and the seeming hesitance to continue to emphasize conflict alongside coexistence in conservation [4–6]. In this paper, we discuss the dynamics of human-wildlife conflict and coexistence, aiming to show the importance of both terms in achieving conservation success.

To eliminate any confusion around the central themes of this paper we define human-wildlife conflict (HWC) as the “spatial and temporal (proximity) of human and wildlife activities where humans, wildlife, or both are affected (negatively)” [7 p80]. Conflicts are negative interactions between humans and wildlife (hereafter referred to as direct interactions), or among people over wildlife (hereafter referred to as underlying social interactions) [3,8]. They often refer to the real or perceived “threats to the economic, health and safety, and psychological well-being of people” [9 p525,^10^] as well as the impacts humans have on wildlife [11,12]. Defining human-wildlife coexistence (hereafter coexistence), however, is more challenging.

Coexistence is understood in many ways [see 13,14], as a term, a behavioral descriptor [11], a set of behaviors [11], a framing approach [3], and a conservation goal [15], among others. From a critical social science perspective, the movement toward convivial conservation has been associated with coexistence literature [16]. Convivial conservation refers to “a vision, a politics, and a set of governance principles that realistically respond to the core pressures of our time’ by promoting ‘radical equity, structural transformation, and environmental justice” [17 p268]. While we acknowledge the importance of such transformative shifts, our research focuses more on coexistence from a pragmatic and applied sense, while attending to deeper structural dimensions whenever possible. For this paper, we ascribe Pooley et al. [18 p784] general definition of “a sustainable though dynamic state in which humans and wildlife coadapt to share landscapes, where human interactions with wildlife are effectively governed to ensure wildlife populations persist in socially legitimate ways that ensure tolerable risk levels.” These definitions are offered to share the authors general thinking”. These definitions are presented to convey the authors’ overarching perspectives on the concepts of conflict and coexistence. However, as this paper explores, the meanings of these terms can vary significantly based on the nature of human-wildlife interactions and the social-ecological contexts in which they arise.

The rise in coexistence literature has galvanized support and coexistence framing has produced a more holistic, optimistic view of understanding human-wildlife interactions, identifying sustainable ways to promote the cohabitation of people and wildlife [3,19]. While this newfound emphasis on coexistence has reshaped much of the research landscape around human-wildlife interactions, it has not come without criticism. Most critiques have pointed out the blind spots of coexistence literature (i.e., inadequate attention to conflict), naïveté of the realities of coexisting with wildlife, and confusion around the terminology and actionability of this focus for conservation researchers and practitioners [5,18,20]. Although other critiques exist, the three identified above are most pressing and deserve more attention.

Our aim in this paper is to explore these concepts and their related critiques through the lens of a recently concluded research project on human-wolf interactions in the Pacific Rim National Park Reserve region of British Columbia, Canada [see 15]. The objectives and findings of this project are well-positioned to offer insight into the definitions, blind spots, and utility of coexistence in research and practice. We begin by describing the three main critiques of coexistence critiques in greater detail, followed by an overview of the study area and methods. Next, we present our research findings. Building on these results, we introduce the human-wildlife interaction matrix (HWIM), a collaborative tool designed to visualize the behavioral and cognitive (and affective) aspects of human-wildlife interactions to support conservation planning. Finally, we offer insights on how future work can deepen the concept of coexistence in conservation and its practical use in planning for, and managing, human-wildlife interactions.

### Critique of human-wildlife conflict and coexistence terminologies

#### Forgetting about “conflict”.

Since the shift toward coexistence in conservation social science, there has been a noted decrease in attention to “conflict” as a key part of human-wildlife interactions [5,18]. For years, scholars have criticized the longstanding conflict bias in how we frame and understand human-wildlife interactions [2,19], only to develop a similar trend toward a coexistence bias in the current literature. The benefits of coexistence-framing in conservation are clear [3,19], but coexistence should not be divorced from the need to understand the realities of the conflicts and challenges that people face in sharing space with wildlife. As Hill [5 p3] states directly in the title “conflict is integral to human-wildlife coexistence.” Because of the dynamic state of human-wildlife relations, a ““conflict-less” or “peaceful” state is likely unachievable”. We believe that coexistence scholars never intended to exclude conflict from discussions, but this rift may stem from a perceived stigma surrounding conflict, as well as those who conflict with, or over wildlife. Although such normative judgments may be warranted at times, conflict can occur to anyone and any community, and in many cases, occurs to those working hard to coexist with wildlife [5]. Greater emphasis on balancing coexistence framing with discussions of the conflicts that are embedded within the task of coexistence, and the real challenges that real people face in sharing space with wildlife, would be beneficial all-around.

#### Perceived naïveté of coexistence.

There is a level of concern associated with the perceived naïveté of some coexistence literature to the realities of coexisting with wildlife, especially wildlife that regularly impact human livelihood and safety [18,21]. Some have argued that coexistence literature often lacks emphasis on human well-being and dignity and the sacrifices that come with achieving different degrees of coexistence [21,22]. In addition to the material costs of living with wildlife, Thondlana et al. [21] describe the non-material costs, which include lifestyle insecurity, fear, and worry, all of which take a great deal of commitment from community members. In most cases, coexistence means accepting a certain level of conflict associated with sharing the landscape with wildlife. Further, once coexistence is achieved, it is never final [5]. The dynamic nature of coexistence means that we need to better embrace the social and ecological conflicts that extend our understanding of coexistence (i.e., human equity, wildlife ethics). Conditions and context will continue to change over time as human-wildlife interactions change, intensify, or both. This will generate challenges and opportunities in how we translate social and ecological issues into a constant evolution of what it means to coexist with wildlife. Key to doing this, however, is ensuring we are clear and concise in how we define coexistence, both as a state and as behavior.

#### Uncertainty of practicality.

Finally, there is a perceived ambiguity around coexistence, engendering doubt about the practicality and utility of the term [4,14]. There have been many attempts to define coexistence and its scope, leading to confusion about what coexistence really is [4,14,18]. As shown above, coexistence has many meanings, so it is important to clarify the intended meaning in specific contexts. It is also important to clarify that coexistence is not the inverse of conflict. To address concerns around the practicality and actionability of coexistence, more local and contextual emphasis is needed on the nature of coexistence efforts. This means that definitions of coexistence will and should vary by sociocultural context, species, and scale [see 23]. Multiple understandings of human-wildlife coexistence can exist and can be equally valuable. The development of local, contextual definitions (or parameters) of these terms speak more directly to the applicability of coexistence to conservation practice in a given area [15,24]. If the “dynamic state” of positive coexistence is considered a desired goal or outcome of conservation efforts, this means that collaborative, equitable processes are needed to determine what that state looks like, what context it is nested in, and what behaviors can contribute to achieving that outcome. Fiasco & Massarella [25] refer to coexistence as a “buzzword” in conservation and that the depoliticized nature of coexistence efforts “fail to address deeper social, political, and economic drivers of HWC.” While this might be true in some cases, such statements undermine countless coexistence efforts that are placing direct emphasis on collaborative, innovative, and decolonial approaches to human-wildlife coexistence [15,26,27]. In fact, we argue along with others [28], that these types of open, collaborative conservation processes are necessary for the continued evolution of our understanding of coexistence and push toward a more equitable and ethical situation for both people and wildlife. This can sharpen our understanding of coexistence as a concept and foster our ability to enact its dimensions toward creating positive shifts in social and environmental well-being.

### Positionality statement

The authors research training and experience related to human-wildlife interactions stretch across social and biological sciences related to human-wildlife interactions and the intersecting social, cultural, and ecological dimensions of interactions [29]. Stemming from our collective research backgrounds and philosophies, this research and the writing of this manuscript are largely influenced by the collaborative and participatory methodological approach. Approaching this work with a participatory worldview means prioritizing the importance of humans as active agents in environmental planning and decision-making. This includes the need to value multiple ways of knowing and being, and producing relevant, practical, and actionable science [30]. This research was carried out under two very broad goals: (1) work toward the well-being and conservation of non-human beings, and (2) ensure human well-being and equity are at the forefront of those efforts. These positionalities and factors largely influenced the decision-making within the research process. All authors align with this standpoint.

## Study area & context

This research was carried out in and around the Pacific Rim National Park Reserve (PRNPR), situated on the southwest coast of Vancouver Island, British Columbia. The Park Reserve is situated on the territories of nine *Nuu-chah-nulth* First Nations (*Ditidaht, Hupacasath, Huu-ay-aht, Pacheedaht, Tla-o-qui-aht, Toquaht, Tseshaht, Uchucklesaht, Yuułu*ʔ*ił*ʔ*ath*․) and two residential communities that serve as tourist hubs for over a million visitors to the region each year (Tofino, Ucluelet) [31]. This highlights the degree of human presence in proximity to the park reserve as well as the diverse interests in the region. Despite the high degree of visitation and recreation, PRNPR is home to stable carnivore populations [15]. The PRNPR area is widely known as a socially, politically, and environmentally progressive place. Paired with an absence of livestock production, the region is well-known for being pro-wildlife and pro-carnivore. Despite this unique scenario, conflicts with wildlife do occur. More recently, conflict with and over wolves have presented challenges to local communities and conservation authorities.

In response to growing human-wolf conflicts in PRNPR, Parks Canada started the Wild About Wolves project [15] in 2018, to reduce human-wolf conflict and work toward coexistence. During the project’s first community workshop in 2018 [15], participants expressed the desire to understand what human-wolf conflict and coexistence means in the region, how locals broadly feel about wolves, and what is driving conflict and coexistence in the region. To do this, we engaged a wide variety of people with interest and experience with human-wolf interactions in the area.

## Methods

Between May 2021 and April 2022, the first author conducted 32 semi-structured interviews, following an interview guide that included both general topics for discussion as well as open-ended questions [32]. Questions and topic probes were designed to inquire about individual’s experiences with wolves and to gain a common understanding of conflict and coexistence across different cultural and situational contexts. The main goal was to learn from community members who are engaged in, and knowledgeable about wolves and wolf conservation, as well as those who are interested and have unique experiences with wolves. Initial interview participants were recruited from a list of individuals previously involved with the Wild About Wolves project provided by PRNPR [15]. This list was then expanded on by using snowball sampling to limit organization bias on the part of Parks Canada. Snowball sampling consisted of asking individuals to recommend other prospective participants during interviews. Doing this, built a stronger, more diverse knowledge base and helped to reach data saturation, whereby no new evidence is being presented [32]. All participants gave verbal consent before partaking in the interview process. This research was approved by the University of Saskatchewan Behavioral Ethics Board (Beh-REB #1839).

Due to the COVID-19 pandemic interviews were held on Zoom or by phone. Interviews ranged from 25 minutes to 124 minutes, with an average time of 57 minutes. Of the 32 knowledge co-producers interviewed, just under 19% identified as First Nations and around 41% as female (Table 1). Most collaborators have lived in the area for over 40 years (31%), and everyone interviewed has lived in the area for at least 5 years (Table 1). Some of these people (12.5%) are currently not residing in the area, but either grew up there and only recently moved away or had worked most of their lives in the area (Table 1). Most of those involved were representing themselves as community members (25%), followed by current or former PRNPR staff (15.6%) and First Nations representatives (15.6%), among other local organizations and industries (Table 1). Out of the 32 total collaborators, 11 were recruited from the initial list provided by PRNPR staff while the other 21 were recruited through the snowball sampling process [33].

**Table 1:** Interview participant demographic breakdown.

Participant breakdown	# of participants
*Gender*	
Male	19 (59.4%)
Female	13 (40.6%)
*Identity*	
Non-Indigenous	26 (81.2%)
First Nations	6 (18.8%)
*Length of residency*	
5–10 Years	4 (12.5%)
11–20 Years	5 (15.6%)
21–30 Years	4 (12.5%)
31–40 Years	5 (15.6%)
> 40 Years	10 (31.3%)
Currently not resident in area	4 (12.5%)
*Primary sector representation*	
Current or Former PRNPR Staff	5 (15.6%)
Local Organization	4 (12.5%)
Tourism Industry	4 (12.5%)
First Nations Representative	5 (15.6%)
Photography/Videography	3 (9.4%)
Government (other)	3 (9.4%)
Community Member	8 (25%)

Interviews were recorded using Zoom Pro and transcribed and coded by the first author using NVIVO 12 Mac software. An initial set of *a priori* codes were developed from a thorough review of the human-wolf interaction literature from 2002–2022. A total of 41 *a priori* codes were identified. An additional 18 codes were identified through the data analysis, bringing the total to 59 codes (or subthemes). While it is beyond our scope to discuss each code in this manuscript, we focus on the codes that specifically fell within the themes of conflict and coexistence. In the results below, we present the contextual definitions created for conflict and coexistence. We asked participants to explain what they believed human-wolf conflict and coexistence meant in the PRNPR to generate these responses. Next, we provide an overview of the actual and perceived cognitive and affective responses to wolves in the region. This was achieved by asking participants how they felt about wolves and how they thought wolves were generally perceived in the area. Then, we provide a breakdown of the many behavioral factors that were prominent in the interview conversations. These span many codes and include both conflict and coexistence behaviors. Last, drawing on behavioral factors, we use examples from the interview results to illustrate how the framework proposed in this manuscript can be applied within collaborative conservation settings.

## Results

### Conflict and coexistence

Of the relevant codes from the interview results eight fell within the conflict category and five in coexistence. The code categories for human-wolf conflict include: (1) attacks and killings, (2) cognitive and affective, (3) food conditioning and habituation, (4) lack of healthy fear/respect, (5) camping, (6) development, (7) photography and videography, and (8) tourism and visitation. The code categories for coexistence include: (1) education, awareness, and communication, (2) cognitive and affective, (3) hazing, (4) Traditional Knowledge and First Nations importance, and (5) programs and projects. Other codes that did not fit within the *a priori* codes emerged from the data. Of the 13 total stated above and shown in Table 2 below, 8 were new codes that did not fit within the *a priori* code list. The themes and smaller subthemes (or codes) help to paint a better picture of what conflict and coexistence looks like in the PRNPR area.

**Table 2:** List of themes and subthemes related to human-wolf conflict and coexistence in the PRNPR regions of British Columbia, Canada.

Themes/Codes	# Files^1^	# Refs^2^
**Conflict/Problem-Oriented**		
Attacks and Killings		
* Wolf attacks on pets*	25	73
* Human aggression toward wolves*	15	28
Cognitive and Affective (Conflict)		
* Fear and perceived risk*	16	29
* Biased and/or negative media*	12	17
* Poor knowledge, beliefs, or judgement*	18	27
* Conflict behavior*	22	42
Other		
* Food conditioning and habituation*	27	109
Little or no healthy fear/respect*	23	37
Camping*	17	42
Development*	13	25
Photography and Videography*	19	44
Tourism and Visitation*	27	81
**Coexistence/Solutions-Oriented**		
Education, Awareness, and Communication		
* Environmental education*	27	83
* General awareness*	24	51
Cognitive and Affective (Coexistence)		
* Positive or neutral attitudes and emotions*	25	50
* Positive/coexistence behavior*	21	42
Hazing*	17	39
First Nations Importance and Traditional Knowledge*	26	94
Programs and Projects*	25	74

New codes that did not fit into the a priori code list are indicated by an asterisk (*). ^1^Indicates the number of unique interview participants who referenced the code/theme, ^2^Indicates the total number of references to that code/theme across all interviews.

#### Conflict.

Participants were mostly unified in their understanding of what human-wolf conflict encompassed in the region, regardless of their diverse backgrounds and associations. During the interviews, participants were asked what human-wolf conflict meant to them. Drawing from the responses, the common themes from responses to these questions, the authors produced the following definition:

*Conflict is the spectrum of human and wolf interactions that turn out negative for one, the other, or both. In this case, conflict is knowingly or unknowingly human-driven and can range from people wanting to be close to wolves to people or wolves being attacked (or killed) to simply wolves becoming habituated to human presence.*

Interview quotes reinforce this definition. A local NGO representative specifically referred to a spectrum in terms of “*…a graduated line in terms of the highest point of conflict is when an animal is destroyed*”, while a former PRNPR staff member acknowledged the diversity of conflicts that can occur by saying “*…there are many forms, and it’s a challenge to deal with all of those various iterations of human wildlife-conflict*.” Participants felt that conflict could start even earlier. As a community member describes “*…if there is a prolonged sense of interest, or that sense of approaching you to discover a little, or they’re curious, or they’re standing their ground…I think that would be a potential risk for conflict*.” When that occurs, a conservation officer warned that it “*starts to present a risk to public safety, or to property, or livestock or animals that are not properly secured.*” Lastly, there were several references to people being at fault and having a responsibility when it comes to conflict. Echoing others, one community member directly stated that “*…we’re aggressors both passively and mindlessly*”, while from another perspective, a *Nuu-chah-nulth Elder* recounted their own personal commitment in saying, “*I view the conflict as being my responsibility, right. The wolf didn’t ask for it, so I think that in conflict, the responsibility becomes ours, that we’re creating this conflict, were not just trying to move away from it*.” These quotes reiterate the human role in preventing and/or dealing with human-wolf conflicts.

Many drivers of conflict were identified in the interviews, spanning a variety of direct and indirect behaviors. The most cited, in order of prominence, were off-leash dogs (off-leash dogs can tempt wolves to pursue them and impact other species) [34], unsecured attractants (i.e., open garbage, human or pet food, littering), seeking out interactions (perception of wolves as not dangerous), direct feeding, economic and tourism development, and photography, among others. Regarding off-leash dogs being the largest contributor to conflict, one community member shared that “*it’s usually ancillary components to human activity, be it chickens, livestock, pets, you know, pets seem to be a big one*.” Someone closely associated with the park added that “*there’s a fairly strong feeling that wolves do not attack people, which probably could be a little less around.*” This wish is understandable but revealing about the perceived utility of fear to shape human behavior, as well as potentially concerning given wolves’ ability to harm pets and people under certain circumstances.

With a popular national park as a key tourist attraction in the region, many participants noted how the added stress of unaware campers contributes a lot to human-wolf conflict and habituation through unsecured attractants. We refer to habituation as the reciprocal process of people and wildlife becoming more comfortable with each other, which has been associated with a greater risk of human-wildlife conflicts and food-seeking behaviors [35,36]. A community member who shares property with wolves stated that careless campers are a big concern. They shared that “*there are people that just don’t take the precautions needed to secure their food…Then, even when food caches [secure food storage devices] are available, people aren’t necessarily using them, whether they’re full, or they’re too far down the beach*.” Photographers and filmmakers also received much attention for their potential role in contributing to conflict. A current PRNPR staff member stated that *“the area has sort of become known as a place where you can photograph wolves, so there’s very well known, professional wildlife photographers, as well as many semi-pro, kind of photographers…and they come here and target those habituated wolves and continue to make it worse, right, and then they kind of feed that cycle of continuing to habituate the wolves, but then also showing what they’re doing on YouTube, or Vimeo, or whatever…so that perpetuates it*.”

These challenges are compounded by the area’s limited geographical space and unceasing development pressure. “*There’s a very thin strip of coastline that’s inhabited by things that wolves eat, and that’s also where the West Coast Trail runs. So, the human-wolf conflict is that they both want to be in the same area, and humans and wolves, as far as I’m concerned, the less they see each other, the better*.” Others felt that development itself in this scenario is driving more conflict. As one *Nuu-chah-nulth* leader shared, “*we make the mistake to think that we’re the superior ones, and disregarding that we live amongst wildlife, and that when we develop into areas, it’s always communicated the other way, you know, that they are the problems. There needs to be recognition to balance*.” The geographical and environmental pressures, along with the other direct drivers mentioned above, are contributing to wolves becoming increasingly habituated and food-conditioned, making them more comfortable around people. This challenge is reinforced by a community member, who stated that “*the danger is the wolves getting habituated… and the danger that follows that is the wolves become aggressive, and then the park shoots them*.” While lethal control of wolves is not a common occurrence in the area, it has occurred an estimated nine times over the past 20 years [37]. In the past, these events have generated tension, between wildlife managers and First Nations, the public, and the broader advocacy community. Taken together, these examples paint a complex picture of human-wolf conflict in the region: our co-developed definition elucidates the key aspects of conflict within the current context.

#### Coexistence.

Participants were largely unified in their understanding of what human-wolf coexistence encompasses in the region, albeit with a greater level and variety of detail than within the conflict definition. In the interviews, participants were asked what human-wolf coexistence meant to them. Drawing from the responses, the common themes from responses to these questions, the authors produced the following definition:

*Coexistence is the mutual, respectful sharing of the landscape between people and wolves in a way that does not compromise the health and well-being of wolves, people, or both. It is an ongoing process in which humans take responsibility for their behaviors and make the necessary personal changes and sacrifices that respect and honor the wolves, keeping them wild and wary.*

Participants reinforced this definition with context and nuance. A local wildlife advocate stated that “*coexistence means that we should be far more respectful of their territory, and if we want the privilege of having animals as part of our families, that we need to be mindful of our neighbors*.” Taking it a step further, a PRNPR staff member stated “…*ultimately, to me, that definition is that these animals can remain on the landscape in a wild manner, without having to run into these issues where they become habituated, and/or food conditioned*.” Interestingly, although the word “coexistence” does not exist in the *Nuu-chah-nulth* language, there is still a shared sentiment about the goals and needs associated with what the term is believed to mean. One *Nuu-chah-nulth* Elder stated “I *think that it’s always really interesting that coexistence, cohabitation, those kinds of things are not in our Nuu-chah-nulth language, right. We share space with someone or something, whether it is human or animal, and they’re always made aware that that’s the way it is, it’s the way it’s always been, it’s always going to be… it’s our way of sharing space*.” Building on this, another community member who lives near wolves, referenced the responsibility of people within this relationship by stating that “*if humans take the time to closely observe and be aware of their surroundings, noticing signs of scat [and] tracks…they already know what is going on. The misunderstanding comes when people don’t think it’s their responsibility to do this and they have not considered any action that may need to be taken to prevent a problem from developing*.” These quotes illustrate how coexistence, in this context, coexistence is largely about sharing space, respect, and responsibility, and how human behavior plays a major role in being able to achieve this outlined definition.

In order of prominence, participants identified fact-based and experiential education, cultural awareness and respect, behavior change (i.e., avoiding interactions, securing attractants), keeping dogs on leash, responsible camping, investing in infrastructure (i.e., wildlife-proof bins, fencing), vigilance, consistent hazing, and policy (i.e., responsible tourism, limiting development) as the key drivers of coexistence in the region.

These drivers were reinforced by contextual quotes from participants. As education and awareness (especially Indigenous Knowledge) came across as the most prominent, a former PRNPR staff member stated that they “*…think that pathway is through education, and knowledge, and field activities, which I think, give people a fact-based, science-based, and perhaps even First Nations-based viewpoint on this animals’ place in nature, and why they’re important.*” Indigenous and non-Indigenous participants each brought up the *Nuu-cha-nulth* principle of “*heshook-ish tsawalk*” - everything is one - as being an important lesson relating to human-wolf relationships. A *Nuu-chah-nulth* leader stated that “*everything in our territory is connected…It’s one of our Chief’s laws, one of our oldest laws*.” Elaborating on the importance of the First Nations viewpoint, another *Nuu-chah-nulth* Knowledge holder added that “*we need to understand that these animals are held in high regard to First Nations people and that interacting with them, in any way, harms that whole existence there because when a wolf gets put down, it literally hurts us personally, as a people*.”

Participants believed that these negative interactions could be avoided by taking the appropriate behavioral steps. Someone closely aligned with PRNRP and who has a lot of experience educating visitors stated “*It’s our responsibility as the sentient ones in this relationship to figure out what the wolves are going to do and work around it…Everybody can get along fine as long as nobody’s trying to actually make friends with the wolf. Like, it’s a wild animal, the less seen of it, the better*.” This notion that it’s desirable to see wolves as little as possible was reinforced by over half of the interview participants as something that is important for coexistence locally. A former PRNPR staff member added that “*to coexist would mean that you would keep your yard and your residence, and anything that might attract these animals at a minimum, manage your dogs to reduce the risk to that animal*.” Doing this requires behavior change from people as one community member stated, “…*coexistence for me, involves sacrifices on the part of the humans or our pets. So, if I know the pack is hanging around, my dogs don’t get walked, because that’s inviting conflict*.” Building on this, another community member stated that “*the biggest problem we have on the west coast, and I’m sure Parks Canada has told you this, is the number of people who do not walk their dogs on-lead. I have heard stories of incidences in the National Park, where people let their dogs run around, and all of a sudden, a couple wolves come out*.”

While local residents and First Nations in the region might be able to strive toward consistency in these behaviors, tourism in the area adds additional challenges. A *Nuu-chah-nulth* Knowledge holder stated that “*…coexistence would have to include acknowledgment of wolves’ areas of preferred areas of travel. Which means, unfortunately for humans that, that we need to restrain ourselves from over-developing foreshores*.*”* There was a lot of discussion about the challenges that tourism presents to coexistence, however, many referenced important shifts in tourism-related mindsets in the area and believe that tourism operators play an integral role in promoting and achieving coexistence. A local tourism operator summarized this very clearly in saying that “*most of the tourist outfits, they speak to their visitors and guests about how to deal with wildlife, and how to be safe and not interact and not interfere with them*.” Building on this, another tourism representative stated that “…*even from a visitor standpoint, when we have people coming from the city where they’ve never seen a wolf. Or they’re coming from Europe where it’s shocking how fascinated Europeans are with the wildlife we have out here. I think that when you have something like an experience to educate somebody in a really engaging or interesting way, they have a newfound respect for whatever it is that they are learning about*.” It was emphasized that tourists are more difficult to control than residents, but it is believed that if everyone does their part, visitors will be more likely to follow by example. Dissecting these complexities of human-wolf interactions in the PRNPR region offers a more comprehensive and contextual foundation for understanding conflict and coexistence.

### Human-wildlife interaction matrix – A tool for collaborative conservation

**Fig 1:**
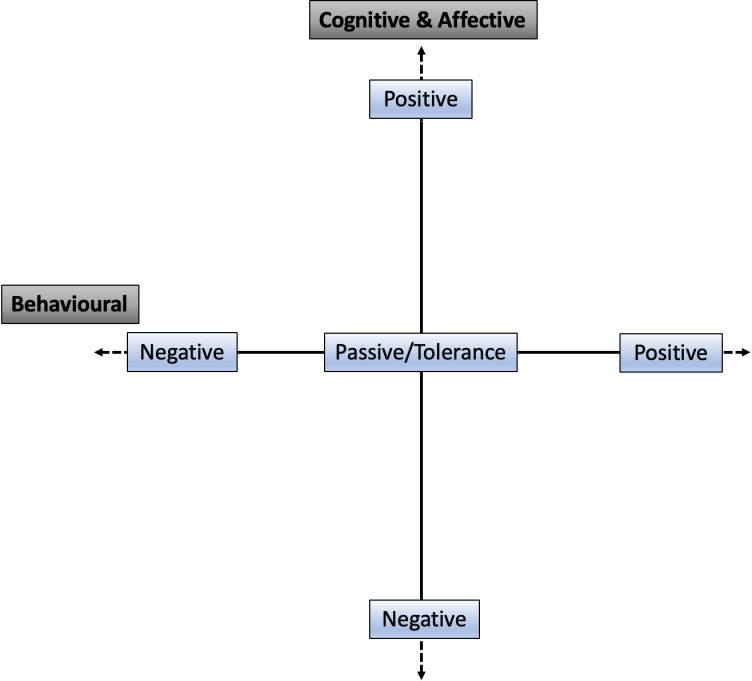
Human-wildlife interaction matrix (HWIM).

As an outcome of the empirical findings of this research, we present the human-wildlife interaction matrix (HWIM) as a collaborative tool for visualizing behavioral and cognitive (and effective) elements of human-wildlife interactions. The HWIM addresses a need in the collaborative conservation space by visually combining aspects of behavioral and cognitive aspects of conflict and coexistence. The matrix comprises a horizontal axis that spans the behavioral elements of HWI [3] as non-fixed ends ranging from negative (left) to positive (right), and a vertical axis that spans the cognitive and affective elements of HWI [4] with non-fixed ends ranging from positive (top) to negative (bottom) (see Fig 1). The HWIM also allows participants to differentiate between direct interactions and underlying aspects associated with the situation at hand. This framework is designed to facilitate open discussion among collaborators in a specific endeavor on the placement of interactions and to help pinpoint more targeted strategies to address conflict. The HWIM is not a “new” framework, but rather a *bricolage*: an extension and blending of existing theory, ideas, and frameworks to better orient toward the particulars of human-wildlife interactions [2,4,11].

Differing from other tools, we offer three main requirements/principles for the use of the matrix:

(1) ***The matrix should be used within an established collaborative research and/or engagement process***. We envision this matrix being used in two ways: (1) within new or ongoing conservation efforts and/or (2) by researchers working as part of ongoing collaborative efforts. Used as a tool within current collaborative efforts, this would involve diverse interest groups and individuals openly and collectively populating the matrix and engaging in discussions about the placement and relevance of the interactions. Once an interaction is placed on the HWIM, the group can then discuss its placement and come to some level of consensus on where the interaction should be on the matrix. As part of the research (shown below), a facilitator, researcher, or group would place interactions on the matrix based on the results of their research and use that as a discussion tool in sharing findings back with the research participants. This allows research participants to offer their thoughts on, and potentially change, the placement of items on the matrix. This is an engaging way to disseminate and extend research findings, but also for participants to see that people share different understandings of HWI, illustrating the need for compromise in plotting and readjusting the placement of points.
(2) ***Use of the matrix should emphasize inclusion and foster open dialogue on controversial or sensitive interactions***. This prioritizes the fact that legacies of injustice and mistrust play a major role in coexistence efforts [38]. This principle works toward building a better understanding of the non-fixed ends of the HWM to include important and emergent trends around inclusive and ethical conservation. This draws on previous calls to move beyond coexistence as an end goal [39] and facilitates space for discussions on the further reaches of more just and equitable coexistence and the social, political, and economic barriers to getting there. This requires the intentional creation of space to discuss these deeper, underlying issues as a means of building more resilient and trusting collaborative institutions for coexistence.
(3) ***The matrix should develop some level of contextual understanding of conflict and coexistence for the HWI being addressed***. Conflict and coexistence mean different things to different people and differ across species and contexts. Clarifying key parameters of these terms as conservation challenges (conflict) and project goals and/or solutions (coexistence) helps to ensure that all collaborators are on the same page. Not all processes will have the time or resources to conduct a research project as presented in this paper, but through facilitated discussions, it is possible to come up with core criteria for what each term is understood to mean in a particular context.

### Applying the human-wildlife interaction matrix

**Fig 2:**
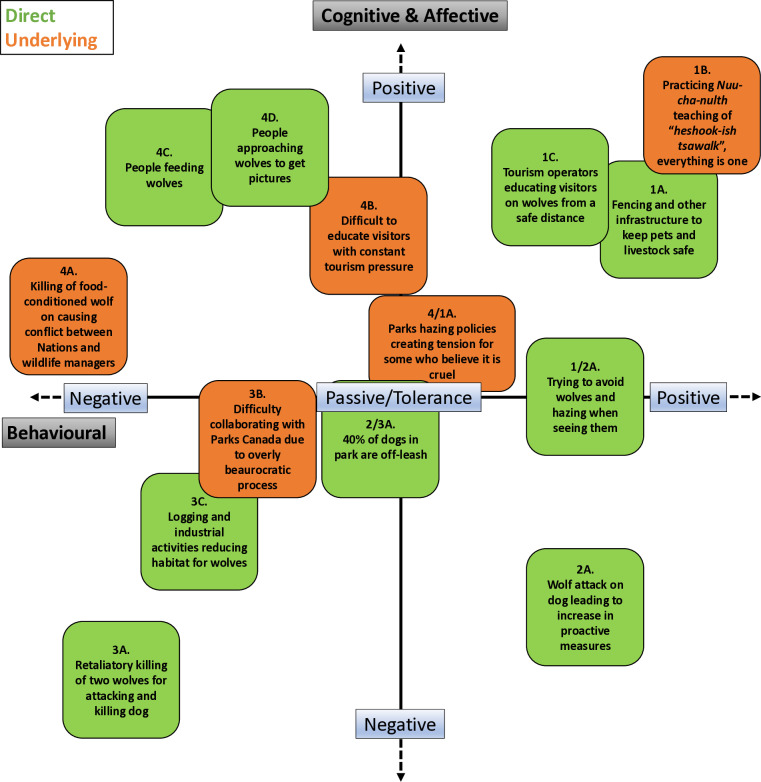
Human-Wildlife Interaction Matrix Framework (HWIM) with placed interactions (Adapted from 3,4).

To illustrate how the matrix can be used, we mapped interactions directly from the interview data onto the matrix to identify relationships between conflict and coexistence, helping to disambiguate and nuance these outcomes (Fig 2). Each quadrant on the matrix is distinct and will necessitate strategies that are unique to those challenges, or groupings of challenges, which can then usefully inform decision-making processes [40]. In the Supplementary Information, we share the full quotes for each of the interactions placed on the matrix to offer more context.

In line with the findings presented in the conflict and coexistence sections, there were very few examples of interactions or instances that could be attributed to negative sentiments toward wolves. The most extreme example was the retaliatory killing of two wolves in 2012 (3A). In contrast, another example of a wolf killing a pet, resulted in the community making positive behavioral efforts (2A). In this instance, it was placed as negative on the cognitive axis due to the assumption that the community was acting out of fear and that there was collective anxiety at the time. Most underlying instances involved Parks Canada. Most notably, the killing of a food-conditioned wolf on *Tla-o-qui-aht* lands by park staff has continued to be a source of tension among some First Nations and the Agency (4A). This instance created conflict far beyond the wildlife side of the interaction. Additionally, collaboration with Parks Canada was considered challenging due to an overly bureaucratic process. While intentions were good, this often sometimes diminished the willingness to participate by some groups or individuals (3B).

There were abundant examples of individuals and groups who feel positively about wolves and whose actions reflected that. These sentiments are demonstrated in many of the expressed coexistence behaviors above, such as tourism operators educating visitors about wolves (1C), local residents and businesses investing in infrastructure to reduce conflict with wolves (1A), and local residents actively avoiding wolves and hazing when they are seen (1/2A). One of the most prominent principles, however, is the *Nuu-cha-nulth* governing law of *heshook-ish tsawalk*, meaning *everything is one*, which guides decision-making and behaviors (1B).

Although positive attitudes are often believed to benefit wildlife conservation [41,42], in this context, it is far more complicated. There were numerous known instances of people approaching wolves (4D) either to have a unique experience or to get a picture, as well as people in the area feeding wolves (4C) for similar reasons. In many cases, these can be visitors, who many participants believe act in ignorance, especially given the difficulty in educating so many visitors in short amounts of time (4B). These behaviors have, however, been observed by both locals and visitors, presenting challenges to coexistence in the region. Displaying even these few examples on the matrix illustrates a clear correspondence with the local understandings of conflict and coexistence detailed above, but pinpoint in a visual way, where tensions and opportunities may exist.

## Discussion

### Conflict and coexistence are contextual, not universal

Our findings illustrate the uniqueness of human-wolf conflict and coexistence in the PRNPR region. Eight of the 13 themes from this research did not fit into the preestablished *a priori* code framework based on the past 20 years of human-wolf interaction research. This may be partially explained by the absence of lifestyle and livelihood activities that are more traditionally associated with human-wolf conflict (i.e., livestock production, hunting). We have also shown that despite widespread positive sentiments toward wolves conflict is still a concern; challenging the idea that coexistence is the absence of conflict, indicating rather that they are interconnected [5]. These observations align with recent calls for more contextual understandings of coexistence and conflict [23]. They also reinforce the idea that conflict is an equally important factor in understanding human-wildlife interactions.

Glikman et al. [39 p447] state that “defining coexistence from the perspective of the individuals and groups…is instrumental to…evaluate if coexistence has been reached and to what degree.” Further, in reference to coexistence, Knox et al. [23], highlight the difficulty of working toward “undefined end goals”. We go further to suggest that conflict is an equally undefined component of human-wildlife interactions. If coexistence, in many cases, is deemed to be a goal, conflict is presumed to be the problem. In our case, the findings show that human-wolf conflict differs from the conventional literature even more so than coexistence, indicating that it is just as contextually driven and deserving of place-based, contextual understandings. Just as working toward “undefined end goals” creates difficulty [23], addressing undefined problems can be equally challenging. Our findings show that generalizations or blanket definitions can lack the necessary nuance to understand both terms in practical, locally actionable ways.

Our co-produced definitions of conflict and coexistence paint a much different picture of human-wolf interactions than those presented in much of the academic literature. In sum, participants emphasized conflict as a spectrum ranging from outcomes that are negative for either people or wolves, that occur to both people who enjoy wolves and those who do not. Participants emphasized that coexistence is an ongoing process revolving around a mutual, respectful sharing of the landscape, including a human responsibility to manage behaviors and make the appropriate personal changes for the good of wolves and people. We believe this level of detail is not unique, rather it is often overlooked by not emphasizing important parameters of coexistence in a given context. We understand not all conservation efforts have the capacity or resources to conduct research projects to help develop these understandings. That said, by ensuring efforts are inclusive and collaborative, facilitators can work toward building a common understanding of these terms as a group. Emphasizing this can reduce confusion and contribute to a more practical and relevant understanding of the terms and their usefulness.

### Coexistence efforts require inclusive, open collaboration

Successful coexistence efforts require inclusive collaboration [28]. This is not a new idea; inclusive and collaborative conservation have gained substantial traction over the past few decades and are needed to ensure the voices of those living with wildlife are heard and acted upon. Our findings reiterate and extend recent claims for conservation efforts that prioritize both human well-being in tandem with biological goals [43,44]. Pooley et al. [18 p785] state that “it is important to build trust and legitimacy and codevelop novel decision-making processes, which take cognizance of different stakeholders’ explanatory frameworks (rational and spiritual), moral frameworks, and risk perceptions.” Participants involved in this project shared personal experiences as well as critical perspectives on the project and the local context of human-wolf interactions and management. This is not always the case in such research, though could be related to the project’s early focus on creating space to discuss underlying social issues associated with the coexistence effort.

Our findings indicate that while positive attitudes alone do not equate to coexistence, fostering a diverse group of knowledgeable, engaged individuals who are treated with dignity and respect can enable an effective space for coexistence planning [45]. Inclusive and collaborative processes that genuinely influence planning and decision-making processes have been shown to improve conservation outcomes by giving local communities a sense of responsibility for their environment [46,47]. That said, participants felt that education and awareness rooted in both Indigenous and Western knowledge are key to coexistence. Determining how these knowledge sources are used, shared, and applied within collaborative spaces is fundamental. While this research, and the broader project, was largely shaped and driven from a Western managerial perspective, special attention was paid to the practice of knowledge use and translation. Practices of “braiding”, “weaving”, or “individualizing” Indigenous and Western knowledge systems in ways that truly complement one another help to avoid the inherent bias toward Western knowledge within these spaces [48–50]. We encourage future research to move beyond this and explore deeper models for knowledge and collaborative equity within cross-cultural conservation settings. Some of these approaches include the plural co-existence model [51], multiple-evidence base [50,52], knowledge co-production [53], and “two-eyed seeing” [54,55], all of which can be applied across multiple diverse forms of knowing. Beyond the direct application, setting up these models of engagement and knowledge use can help to generate space for open discussions on productive tensions that may exist.

The HWIM offers potential benefits to collaborative efforts. First, the matrix acts as an analytical tool that attends to both quantitative and qualitative ways of thinking about human-wildlife interactions. Second, it offers a visualization of the relation between cognitive and behavioral factors, which can offer even more direction in planning for targeted policy and practice interventions. Third, the simplicity and flexibility of the HWIM make it applicable across disciplines, methodologies, and within cross-cultural settings. This is a tool that, when used within the appropriate setting, can facilitate inclusive conservation discussions.

### The HWIM can help disambiguate conflict and coexistence

While many recent frameworks have been influential in the advancement of coexistence theory [3,4,11,13,56], the HWIM builds on these to produce a more adaptable and practical tool for coexistence. The conflict-to-coexistence continuum is the most commonly discussed visualization and has been influential in efforts to re-frame research and practice of human-wildlife interactions [11,39]. Although not the intention of the continuum, we agree with Pooley et al. [18 p785] that it could be “interpreted as suggesting conflict and coexistence occupy opposite poles of a linear continuum.” Bhatia [4] suggests an alternative framing ranging from negative coexistence to positive coexistence, which includes conflict without polarizing the terms within the visualization. The HWIM builds on Frank [11], Bhatia [4], and Bhatia et al. [2] in two main ways: (1) it is designed as an applied tool rather than a theoretical framework, and (2) it differentiates between direct human-wildlife interactions and underlying social interactions that impact, or are associated with a particular human-wildlife interaction. By including cognitive and emotional factors alongside behavioral ones, the HWIM can enable discussion on the relationship(s) between these dimensions of human-wildlife interactions [4]. The HWIM builds on these ideas, creating a more adaptable tool for coexistence dialogue, and planning.

The example application of the HWIM presented above highlights several direct and underlying interactions that are crucial to human-wolf coexistence effort in the PRNPR region. These include tensions around the lethal control of wolves in First Nations territory, difficulty balancing the benefits and challenges of tourism, and challenges managing people and their expectations around wolves. The matrix also highlights the promise of proactive incentive programs, the strength of *Nuu-chah-nulth* knowledge of and respect for wolves, and tourism operators working toward raising awareness on wolf coexistence. While some have criticized the effectiveness of using practical tools as being unable to attend to deeper dimensions of human-wildlife interactions [18], we hope that the HWIM can help to facilitate these deeper conversations. Many frameworks often fall short of addressing deeper issues within conservation. This is likely due to a lack of practical applications for research-driven frameworks. Moreover, successful conservation efforts ought to attend to the underlying social challenges associated with coexistence as much as, or more than, the ecological challenges.

For this paper, the placement of these scenarios on the HWIM was based on the author’s subjective interpretations. The authors intended to present this matrix back to research participants to offer a chance for feedback, change, and further dialogue. Due to funding constraints, this was not able to happen. We hope that the framework can be used as an applied tool for both research and practice to help bridge social and ecological outcomes for coexistence. Further addressing critiques around the practicality and utility of coexistence, the matrix could be thought of, and used, as a tool to help disambiguate conflict from coexistence. We understand that all the intricacies of human-wildlife interactions, do not always fit into a “2 × 2 box”. However, we feel that the matrix can facilitate discussions toward understanding these subtleties for more productive outcomes. We also understand that the case study presented intersects with much deeper issues associated with local political economies, animal agency, environmental ethics and justice, among others. While these topics are beyond the scope of this paper, we hope that future work will explore these factors in ways that build on the findings presented in this paper. Conservation challenges are dynamic and ever-changing, meaning that the processes and tools we use require continued adaptation as contexts change. Although the HWIM framework is intended for use in collaborative settings, the rich interview data on the behavioral and cognitive elements related to human-wolf interactions in the PRNPR area offered a unique opportunity to demonstrate how this tool could be used.

Lastly, the HWIM is not intended to be a prescriptive tool, but rather to offer an opportunity for open dialogue on the placement of interaction components, and a chance to visualize their own understanding of conflict and coexistence, including issues of justice, ethics, and equity [44,57]. In the final chapter of their book, Frank et al. [3] clarify that conflict and coexistence are not, and should not, be fixed ends of a spectrum and that emerging issues extending our understanding of both terms should continue to push in that direction. Some of these emergent needs include greater emphasis on equity and well-being among those who share space with wildlife [44] as well as wildlife empathy [12] and the ethical treatment of wildlife [58]. These are growing areas of research focus that can extend our understanding of conflict and coexistence.

## Conclusion

Our co-produced understanding of both conflict and coexistence in the context of human-wolf interactions in the PRNPR region has not only advanced local coexistence efforts but also demonstrated the practical utility of collaborative, community-directed coexistence research in conservation practice. Building on these outcomes, we stress two important distinctions that are supported by our findings. First, conflict and coexistence are interrelated, and contextual, and deserve to be understood as such. Second, collaboration is necessary, but insufficient by itself to ensure coexistence. Real efforts for inclusion and trust-building must be made to generate a strong local desire to coexist and help to build broader coexistence practices. Though we emphasize the uniqueness of this case study, these broader lessons and the HWIM tool are adaptable to a far greater range of human-wildlife interactions. We demonstrated how the HWIM can help develop and refine local understandings of conflict and coexistence. Using this matrix can facilitate open, honest discussions on direct and social tensions around human-wildlife interactions, and more broadly, help to disambiguate and increase the pragmatic value of human-wildlife coexistence as a conservation goal.

## Supporting information

S1 File Direct interview quotes related to the corresponding interactions placed on the Human-Wildlife Interaction Matrix (Fig 2). (DOCX)
