# Supplementary material for: Broadening the spectrum of conflict and coexistence: A case study example of human-wolf interactions in British Columbia, Canada
Source: PLoS One. 2025 Feb 7;20(2):e0318566. doi: 10.1371/journal.pone.0318566 (PMC11805382; doi:10.1371/journal.pone.0318566)
Supplement: S1 File — (DOCX) [file pone.0318566.s001.docx]

**Supporting Information**

Table 1. Direct interview quotes related to the corresponding interactions placed on the Human-Wildlife Interaction Matrix (Figure 2)

| **Number** | **Associated Quote** |
| --- | --- |
| 1A | *“…we have chickens as well, so there's the eight-foot fence, and then within that, is a smaller chicken coop, which also has plywood along the side of the outdoor runs, so that the wolves can't see them.” (#22)* |
| 1B | *“We really go by the teachings of heshook is tswalk (everything is one), and interconnected. So, there is no us and them, it is, you know, it's a grouping, we are all kind of one.” (#28)* |
| 1B | *“…wildlife tour operators, they're all focused on conservation…when there are wolf sightings during a trip, it's usually from a boat… the boat driver will slow down enough just for someone to take that as a moment of acknowledgement that that's what they're looking at, but also they take it as an opportunity to educate the visitors that they are taking out.” (#8)* |
| 1/2A | “I don’t try to get close, I never did, and if they got close to me, then they got a reminder that people are bad, and chase them away.” (#17) |
| 2A | *“….a dog was attacked… really badly injured. Shortly after started seeing residents building like 6-foot-high fences, solid wood fences, around their yards so it's great to see those kinds of responses, where it's not just “shoot the wolves”, you know” (#3)* |
| 2/3A | *“Park research and monitoring data shows that there have been upwards of 100,000 dogs on the beaches in the Long Beach unit over the course of a season… upwards of 40,000 of those dogs were running at large and were very vulnerable to being preyed on.” (#3)* |
| 3A | *“There was a case here where a wolf killed a small dog, and then one of the family members managed to shoot two wolves later on and threw the bodies in a dumpster…”(#1)* |
| 3B | *“…there are so many checks and balances and bureaucratic protocols that they [Parks] have to go through, that it strips it down from what it’s intended to be…” (#8)* |
| 3C | *“there's a lot of logging, which is highly contentious on the island right now…So, I think that they’re [wolves] being impacted in their range…their having to go and look in different places and find different sources…” (#31)* |
| 4A | *“It [wolf] was destroyed on Tla-o-qui-aht lands and it really caused this whole conflict, and giving voice to a whole different view, it really raised the Tla-o-qui-aht view that, in fact, this is this is breaking natural law, this is not how we want to conduct ourselves, have to have wolves destroyed is absolutely unacceptable.” (#20)* |
| 4B | *“…the tourists are just constant, it’s always different people, every single one of them needs to be educated…and I'm not sure how much people retain.” (#18)* |
| 4C | *“…multiple different people have fed wolves here.” (#29)* |
| 4D | *“There’s more and more a culture of people thinking it’s okay, or not knowing better, around approaching wolves or allowing wolves to approach them, or wanting to get pictures and that kind of think, and I think even more so now with the rise of social media and sort of the desire to get likes and followers...” (#29)* |
| 4/1A | “…in the parks, both provincial and national parks, we encourage hard hazing, like throw sand and sticks at animals that come towards you. When we first started to get out with that messaging, there was some pushback from the public. They felt like we were being cruel, you know, but it's like, yeah, it might seem cruel, but you're actually saving those animals if you can…reinforce that if they approach humans, that that is not a good thing for them” (#13) |
